# Re: Ureteric injury during gynaecological surgery – Lessons from 20 cases in Canada.

**Published:** 2021-03-31

**Authors:** GP Jacob, GA Vilos, F Al Turki, G Bhangav, B Abu-Rafea, AG Vilos, A Ternamian

**Affiliations:** Department of Obstetrics and Gynecology, Chatham-Kent Health Alliance, Chatham, Ontario, Canada;; Department of Obstetrics and Gynecology, Western University, London, Ontario, Canada;; Department of Obstetrics and Gynecology, King Fahad Medical City, Riyadh, Saudi Arabia;; Department of Obstetrics and Gynecology, University of Toronto, Toronto, Ontario, Canada.

Dear Editor,

In our publication (Jacob et al., 2020) we stated on page 39, paragraph 4, the following regarding why gynaecologists avoid cystoscopies: “The reason for this is that acquiring cystoscopy skills is not part of the Obstetrics and Gynecology residency curriculum for Royal College of Physicians and Surgeons of Canada (RCPSC) certification and most residents graduate from their obstetrics and gynaecology training without learning to perform a cystoscopy and do not feel comfortable using this basic and fundamental diagnostic instrument”.

This statement is erroneous. Recently, it was pointed out to us that the Royal College of Obstetricians and Gynecologists of Canada Objectives of Training in the Specialty of Obstetrics and Gynecology were amended and include the following statement applicable after July 1, 2016:

“The following procedures in List A are those that the fully trained resident in Obstetrics and Gynecology must be competent to perform independently: 5.2.2.41. Limited cystoscopy”

We regret our error and apologise for any inconvenience.

G.P. Jacob, G.A. Vilos, F. Al Turki, G. Bhangav, B. Abu-Rafea, AG.. Vilos, A. Ternamian 
E-mail: gjacob2010@gmail.com
